# Myoelectric Arm Orthosis Assists Functional Activities: A 3-Month Home Use Outcome Report

**DOI:** 10.1016/j.arrct.2023.100279

**Published:** 2023-07-13

**Authors:** Sarah R. Chang, Nicole Hofland, Zhengyi Chen, Curtis Tatsuoka, Lorie G. Richards, Margaretta Bruestle, Harry Kovelman, Jonathan Naft

**Affiliations:** a Orthocare Innovations, LLC, Edmonds, WA; b Myomo, Inc, Boston, MA; c Case Western Reserve University, Cleveland, OH; d University of Pittsburgh, Pittsburgh, PA; e University of Utah, Salt Lake City, UT; f Geauga Rehabilitation Engineering, Inc, Chardon, OH

**Keywords:** Activities of daily living, Orthosis, Rehabilitation, Stroke, Upper extremity

## Abstract

**Objective:**

The objective was to compare task performance in individuals with upper limb impairments with and without a myoelectric arm orthosis.

**Design:**

Three-month observational study. Participants met at 4 time points after receiving their myoelectric orthosis (2-Weeks, Month-1, Month-2, Month-3) to complete 4 standardized common daily tasks.

**Setting:**

Nationwide sessions completed remotely over videoconference calls at home. There were no specific clinic affiliations.

**Participants:**

Adults with upper limb impairment due to stroke who were in the process of being fit with a myoelectric arm orthosis as a first-time user.

**Interventions:**

The orthosis was a custom-fabricated myoelectric arm orthosis called the MyoPro®.

**Main Outcome Measures:**

Functional tasks were completed at each session with and without the MyoPro. Participants were evaluated on their success and the time required to complete each functional task. Longitudinal mixed and longitudinal mixed logistic regression models were analyzed.

**Results:**

Eighteen individuals with chronic arm weakness due to stroke were included in the analysis. Statistically significant and clinically meaningful improvements were observed on the functional tasks in the participants’ homes. By 3 months, participants successfully used the MyoPro to accomplish the tasks, reduced the amount of time spent to complete the tasks, and had a higher probability of success as compared with at 2 weeks. With the MyoPro, participants showed significant improvement in overall task completion and completed the tasks in a significantly decreased time as compared with without the MyoPro.

**Conclusions:**

The MyoPro provides a stabilizing support to the weak arm of individuals after stroke and enables individuals to use their impaired arm to complete functional tasks independently in the home environment.

## Highlights

• Myoelectric arm orthosis supports ability to do functional tasks in the home.
• MyoPro orthosis actively assists individuals with upper limb impairment post-stroke.
• Evaluated users with set of standardized tasks simulating daily living activities.
• Individuals completed tasks faster and more successfully with MyoPro than without.

Stroke is the leading cause of disability in the United States,^1^^,^^2^ leaving up to 80% of individuals after a stroke with full or partial unilateral paralysis.^3^ Upper limb hemiparesis can severely limit a person's ability to use their affected side to accomplish activities of daily living and instrumental activities of daily living (I/ADLs; eg, self-dressing, feeding, laundry, or cooking). Most recovery of upper limb function occurs within the first 3 months after stroke; fewer than 15% of individuals restore normal motor function of the paretic upper limb.^4^, ^5^, ^6^

Hemiparesis causes 2 barriers to function: joint instability and reduced ability to activate muscles in the correct pattern and force for task completion. There are existing rehabilitation approaches designed specifically to help individuals with hemiparesis, but these interventions, such as therapy sessions, typically occur in the acute phase and are limited due to insurance payment policies.^7^ There are very few options available to address restoration of arm and hand function, especially for individuals with chronic stroke (over 6 months post-stroke).^8^^,^^9^ Traditional rigid braces have been used to reduce joint instability, but these usually limit upper extremity function and do not promote functional movement.^8^

A newer approach using myoelectric bracing to support and rehabilitate a hemiplegic upper extremity called the MyoPro® has shown to have an effect on function outside the acute phase of stroke recovery.^10^^,^^11^ The MyoPro is a custom-fabricated myoelectric arm and hand orthosis^a^ designed to restore function in individuals with upper limb impairment by providing joint motion assistance and rigid support to stabilize the weak arm as it is moved.^12^^,^^13^ The MyoPro uses surface sensors to measure volitionally-generated electromyography signals in the muscles of the paretic upper limb and uses the signals to control actuator-applied assistance of elbow flexion/extension and opening/closing the hand. The MyoPro can be used at home as it promotes functional movement, providing opportunity for neuro re-education during daily tasks. Therefore, it has the potential to support motor learning through increased opportunities for task practice and to allow functional task completion even when post-stroke recovered motor control remains significantly impaired.^10^ Prior MyoPro research in individuals with upper limb weakness has shown improved motor control,^11^^,^^13^^,^^14^ user-reported function, and independence in people with chronic stroke even after engaging in standard rehabilitation therapies prior to using the MyoPro.^15^, ^16^, ^17^

The purpose of this research was to evaluate the use of the MyoPro in assisting with daily task completion, rather than as a training tool. Therefore, we assessed performance in standardized functional tasks with and without (w/wo) the MyoPro in a cohort of individuals with post-stroke hemiparesis. To address limitations in prior studies, we observed participants in their own home environment over 3 months and evaluated basic functional gross motor movements relevant to the MyoPro's capabilities.

## Methods

This was a prospective single arm cohort observational study. Participants meeting study criteria were fit with a custom MyoPro orthosis. After fitting, participants completed research sessions on a regular basis. Therapy was encouraged, but MyoPro learning and progress were based on the individual care received. No research activities involved MyoPro training.

### Participants

Individuals with hemiparesis who were being fit with the MyoPro (w/MyoPro) as first-time users were recruited nationwide from Myomo, Inc's patient database. All participants provided written informed consent before participating in the Institutional Review Board approved study (WCG IRB #20211799).

The main inclusion criteria were adults with upper limb motor impairment caused by stroke; medically stable; ability to generate detectable electromyography signals to operate the MyoPro; and adequate passive range of motion in the shoulder, elbow, wrist, and fingers (see supplemental text S1 [available online only at http://www.archives-pmr.org/] for all inclusion/exclusion criteria).

### Experimental protocol

After providing consent, participants completed data collection sessions in their home remotely over videoconference. Before receiving their MyoPro, a baseline session evaluated their performance on a battery of functional tasks with their paretic side. After receiving their MyoPro, 4 sessions occurred where the battery of tasks was completed w/wo the MyoPro: 2-weeks (2-Weeks), 1 month (Month-1), 2 months (Month-2), and 3 months (Month-3).

### Battery of functional tasks

**Fig 1:**
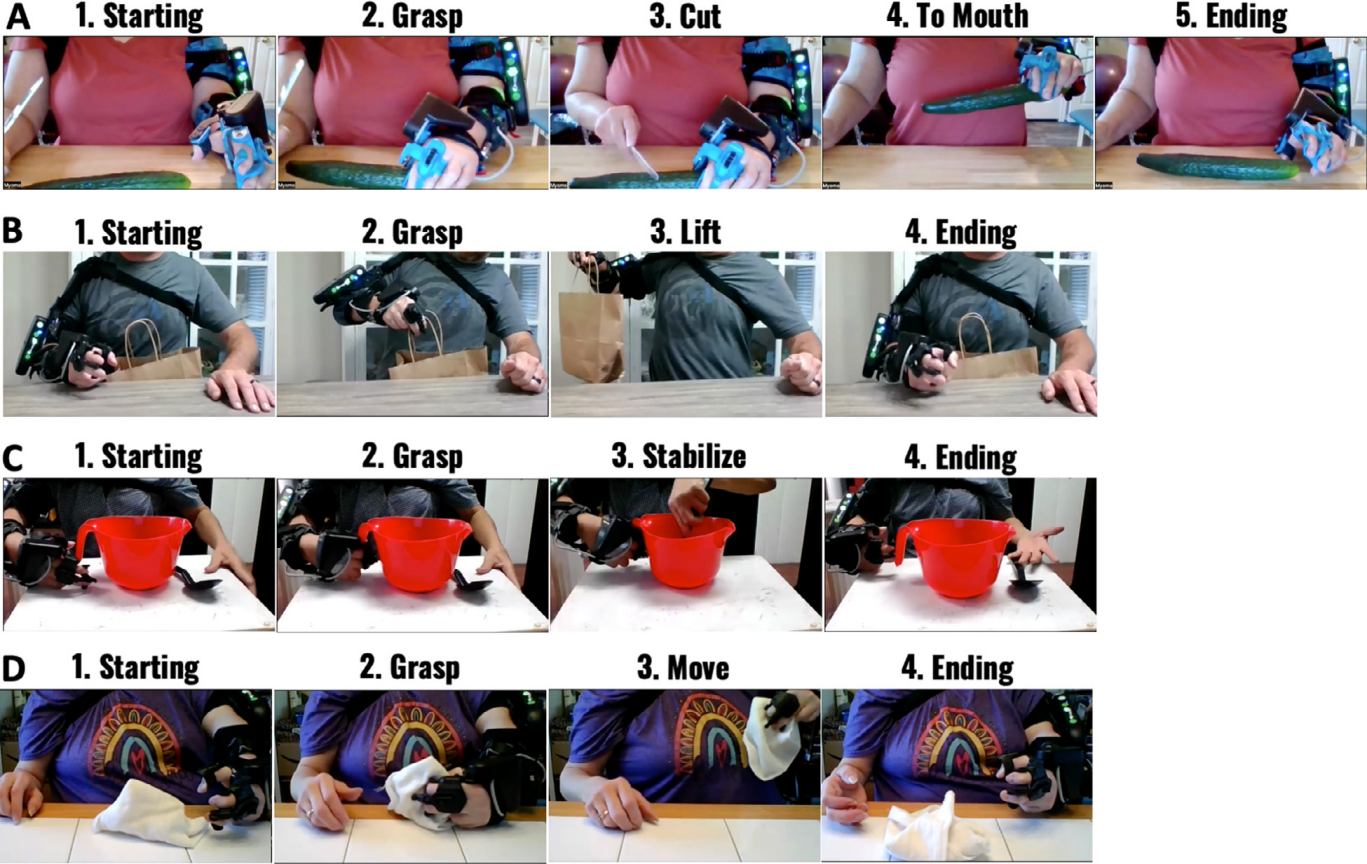
Each component of the tasks shown by a participant. (A) Pickle, (B) Bag, (C) Bowl, and (D) Towel.

A set of pre-identified functional tasks that simulate common I/ADLs that are applicable to the MyoPro's capabilities of grasp/release and elbow flexion/extension were selected. This builds upon prior work reported by Peters et al.^18^ The tasks included the following: move object to mouth (fig 1A), hold object in space (fig 1B), stabilize object (fig 1C), and move object to a new location (fig 1D). The completion order of the tasks was randomized in each session to reduce bias due to order.

For each task, total completion time and overall success in completing the entire task and each of its components were analyzed. Each task had multiple components that were scored (0: unable to achieve, 1: able to achieve) and timed (maximum of 45 seconds allowed per component attempt). The component scores and times were summed for a total score and total time to complete each task. If a participant scored a zero on a component, the time was recorded as 45 seconds and all subsequent components were also scored with zeros. Participants were not informed that they were being scored and timed to reduce apprehension bias. The battery of tasks was evaluated by 1 of the trained raters (S.C., N.H., M.B.) at each session (supplemental text S2 [available online only at http://www.archives-pmr.org/] for additional instructions).

### Statistical analysis

Baseline characteristics were described using mean ± SD or frequency (%). For the time to complete a task at a given time point, we summed the total time taken for all task components and for the w/wo MyoPro scenarios for each participant. Longitudinal linear mixed effects models^19^^,^^20^ were fit to model the trajectory of change of w/wo MyoPro total time difference (w/wo-TtimeDiff) from the first time point (2-Weeks). Mixed models were also chosen as a robust approach to account for any missing data that were missing at random. The w/wo-TtimeDiff was calculated by subtracting the total completion time without the MyoPro (w/o-MyoPro) from the total completion time w/MyoPro at each time point. The change of w/wo-TtimeDiff at each of the time points from the 2-Weeks time point was then calculated by subtracting the total time difference at 2-Weeks from the total time difference at each time point. The change of w/wo-TtimeDiff at each of the monthly time points from 2-Weeks was modeled for all 4 tasks. Two-sided significance level was set at 0.05. For post hoc analyses comparing changes of w/wo-TtimeDiff to zero for each of the different time points, *P* values were adjusted for multiple testing using the Holm-Bonferroni correction. Longitudinal models included fixed effects for time (Month-1, Month-2, and Month-3), as well as the first time point (2-Weeks) w/wo-TtimeDiff value, and random effects for participants to account for within-subject correlation. Other potential confounders were considered for inclusion as covariates: age, sex, race, years since diagnosis before received MyoPro, whether the participants’ dominant side was treated, whether participants generally request help everyday or not everyday with I/ADLs, number of times the participants worked with professional therapy per month (0, 1-5, 6-10), and the completion order of the tasks. Inclusion of a covariate in multivariate setting was based on their univariate analysis *P* values with cutoff of 0.05. Covariance structure selection that reflected serial autocorrelation was based on the model fit statistic -2 Res Log Likelihood. Models considered included first-order autoregressive model, first-order heterogenous autoregressive, and first-order Toeplitiz. Analyses on task completion time were performed using SAS Software^b^.

For task component completion status (yes/no), we first applied McNemar's test on each task component comparing w/wo MyoPro. Then, longitudinal mixed logistic regression models were fit to analyze the effect of w/MyoPro compared with w/o-MyoPro and to model this effect across all time points for each of the task components, adjusted for the corresponding completion status of each task's first component w/MyoPro at the first time point. Random effects for subjects were included as well. Other confounders were considered for inclusion in a model as described above. *P* values were adjusted for multiple testing of components within each task using the Holm-Bonferroni correction. Analyses for the status of task completion were performed using R 4.2.1 Software^c^.

## Results

Twenty-five individuals with chronic stroke were enrolled in the study. However, 18 individuals were analyzed, because 7 individuals were excluded from analysis due to at least 3 skipped sessions or withdrawing from the study before Month-3 (table 1). Of the 18, 5 participants were missing 1 time point dataset, and 1 participant was missing 2 time point datasets. In a baseline evaluation before receiving the MyoPro, all participants (except #12) were unable to complete the battery of tasks with their paretic arm w/o-MyoPro.

**Table 1:** Participant characteristics

	Age	Sex	Race	Affected Arm	Dominant Arm	Years Post Injury to MyoPro Delivery	I Require Help for…*
1	46	F	White	Left	Right	3.7	Just a few activities every day
2	35	M	White	Left	Right	3.9	Most activities every day
3	48	M	White	Right	Right	5.0	Only occasional activities, but not every day
4	67	M	Asian or Pacific Islander	Right	Right	10.9	Only occasional activities, but not every day
5	52	M	White	Right	Right	0.8	Just a few activities every day
6	28	M	White	Right	Right	4.9	Just a few activities every day
7	61	M	White	Right	Right	1.1	Only occasional activities, but not every day
8	67	M	Black or African American	Left	Right	1.6	Just a few activities every day
9	71	M	American Indian or Alaska Native	Right	Right	4.3	Just a few activities every day
10	52	F	White	Right	Right	4.8	Only occasional activities, but not every day
11	50	F	Black or African American	Right	Right	5.1	Only occasional activities, but not every day
12	62	M	White	Left	Right	5.9	Only occasional activities, but not every day
13	65	M	White	Left	Left	4.9	Only occasional activities, but not every day
14	36	F	White	Left	Right	4.5	Only occasional activities, but not every day
15	52	M	White	Left	Left	8.7	Just a few activities every day
16	21	M	White	Right	Right	3.7	Just a few activities every day
17	66	M	White	Right	Right	6.8	Only occasional activities, but not every day
18	66	F	White	Left	Right	3.0	Just a few activities every day
**Mean (SD) or count (%)**	52.5 (14.7)	F(5 (27.8)), M(13 (72.2))	White 14 (77.8)	Left(8 (44.4)), Right(10 (55.6))	Left(2 (11.1)), Right(16 (88.9))	4.6 (2.5)	Every Day(50.0), Not Every Day(50.0)

⁎ Participants were asked to subjectively report their perception of performance and engagement in daily activities. Participants were not required to specify if they completed daily activities with their affected arm or not.

### Changes in the Total Time to Complete the Functional Tasks w/wo MyoPro

#### Pickle: move object to mouth

**Fig 2:**
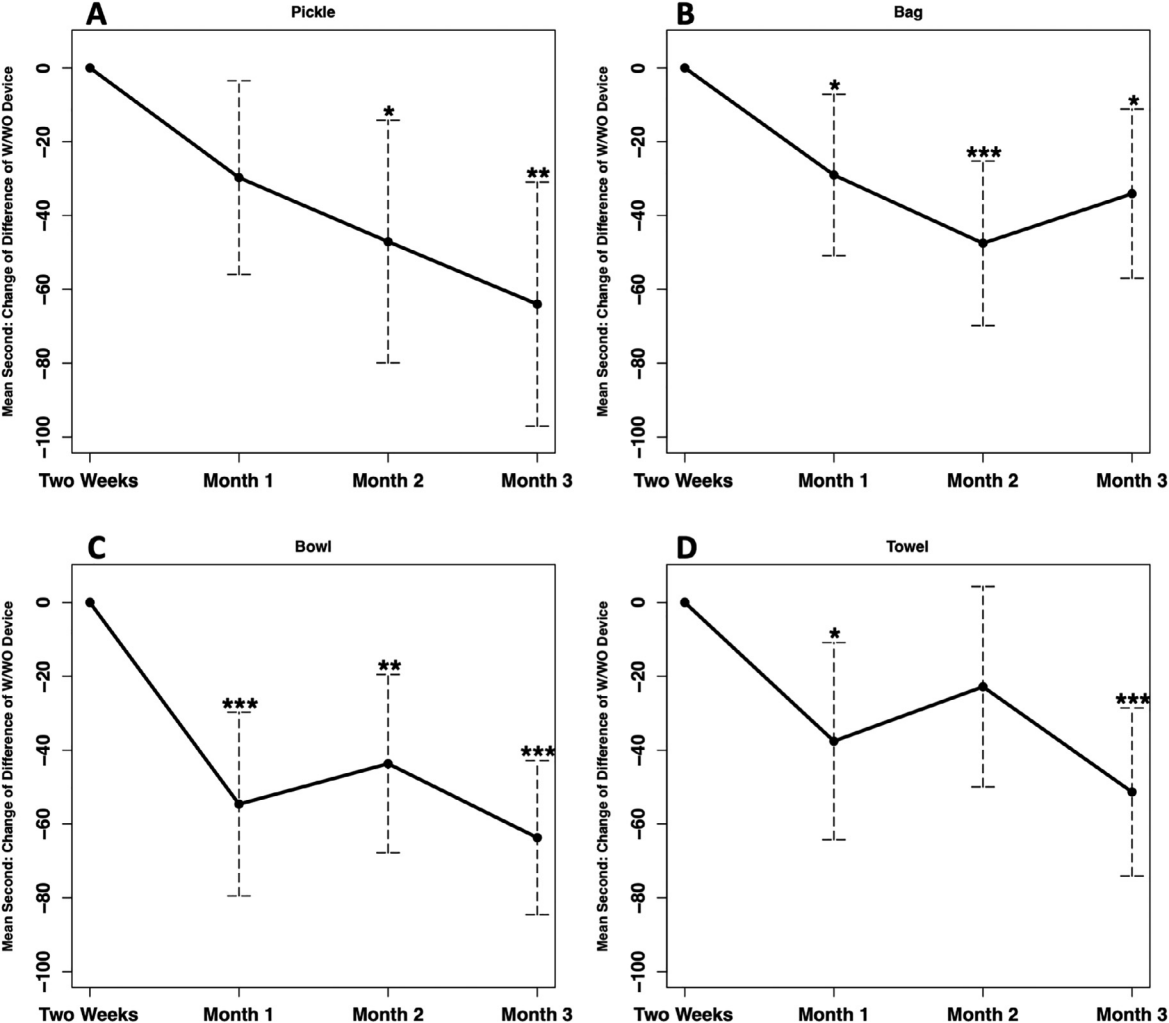
Mean change from 2-Weeks of w/wo MyoPro total time difference for the tasks over the time points. (A) Pickle, (B) Bag, (C) Bowl, and (D) Towel tasks. Results of longitudinal linear mixed effects model adjusted for the first time point (2-Weeks) w/wo total time difference value and other chosen covariates. The variable, w/wo-TtimeDiff, is a measure of the difference between the time taken with and without the MyoPro changes across the sessions, where a larger negative value indicates that the participant was relatively quicker performing the task with the MyoPro than without. *adjusted *P*<.05; ^⁎⁎^adjusted *P*<.01; ^⁎⁎⁎^adjusted *P*<.001.

With the MyoPro, the mean time to complete the pickle task improved by 67.5 seconds from 2-Weeks to Month-3 (supplemental table S1, available online only at http://www.archives-pmr.org/). Without the MyoPro, there was little change in the mean time from 2-Weeks to Month-3. Statistically significant changes from 2-Weeks for the w/wo-TtimeDiff were observed by Month-2 (adjusted *P*=.020) and Month-3 (adjusted *P*'s=.0015, table 2, fig 2A). A smaller difference between completion time in the w/wo conditions at 2-Weeks was associated with larger differences between completion time in the w/wo conditions at each of the following time points [estimate (95% confidence interval [CI])=-0.45 (-0.70, -0.20), *P*=.0009].

**Table 2:** Mean (SD) change from 2-Weeks of w/wo MyoPro total time difference for all tasks

	Month-1	Month-2	Month-3
Pickle	-32.8 (56.3)	-47.1 (71.7)	-58.2 (76.8)
Bag	-29.1 (42.8)	-46.7 (57.8)	-28.3 (43.2)
Bowl	-56.7 (50.8)	-43.3 (57.4)	-63.9 (46.0)
Towel	-44.2 (58.5)	-33.1 (70.2)	-53.2 (57.3)

#### Bag: hold object in space

With the MyoPro, the mean time to complete the bag task improved by 36.2 seconds from 2-Weeks to Month-3 (supplemental table S1, available online only at http://www.archives-pmr.org/). Without the MyoPro, there was little change in the mean time from 2-Weeks to Month-3. Statistically significant changes from 2-Weeks for the w/wo-TtimeDiff were observed by Month-1 (adjusted *P*=.034) and Month-2 (adjusted *P*=.0006). Mean change between 2-Weeks to Month-3 was significant for the w/wo-TtimeDiff (adjusted *P*=.015, table 2, fig 2B). However, the mean change between 2-Weeks to Month-3 was less than between 2-Weeks to Month-1 and -2. A smaller difference between completion time in the w/wo conditions at 2-Weeks was associated with larger differences between completion time in the w/wo conditions at each of the following time points [estimate (95% CI)=-0.42 (-0.74, -0.10), *P*=.012].

#### Bowl: stabilize object

With the MyoPro, the mean time to complete the bowl task improved by 65.4 seconds from 2-Weeks to Month-3 (supplemental table S1, available online only at http://www.archives-pmr.org/). Without the MyoPro, there was little change in the mean time from 2-Weeks to Month-3. Statistically significant changes from 2-Weeks for the w/wo-TtimeDiff were observed by Month-1 (adjusted *P*<.001) and continued to improve after Month-1 through Month-3 (adjusted *P*’s<.01). Mean change between 2-Weeks to Month-2 was significant for the w/wo-TtimeDiff (adjusted *P*=.003, table 2, fig 2C). However, the mean change between 2-Weeks to Month-2 was less than between 2-Weeks to Month-1 and -3. A smaller difference between completion time in the w/wo conditions at 2-Weeks was associated with larger differences between completion time in the w/wo conditions at each of the following time points [estimate (95% CI)=-0.46 (-0.69, -0.23), *P*=.0003].

#### Towel: move object to a new location

With the MyoPro, the mean time to complete the towel task improved by 60.8 seconds from 2-Weeks to Month-3 (supplemental table S1, available online only at http://www.archives-pmr.org/). Without the MyoPro, there was little change in the mean time from 2-Weeks to Month-3. Statistically significant changes from 2-Weeks for the w/wo-TtimeDiff were observed by Month-1 (adjusted *P*=.023). From 2-Weeks, the w/wo-TtimeDiff was not significant at Month-2 (adjusted *P*=.29) but was significant at Month-3 (adjusted *P*<.001, table 2, fig 2D). A smaller w/wo-TtimeDiff at 2-Weeks was associated with larger change of w/wo-TtimeDiff at each of the time points after 2-Weeks [estimate (95% CI)=-0.56 (-0.81, -0.30), *P*=.0001]. When compared with those who used the MyoPro on their non-dominant arm, participants who used the MyoPro on their dominant arms exhibited larger changes in w/wo-TtimeDiff at each of the time points after 2-Weeks [estimate (95% CI)=-48.0 (-87.5, -8.6), *P*=.019].

### Success in completing the components of the tasks

**Fig 3:**
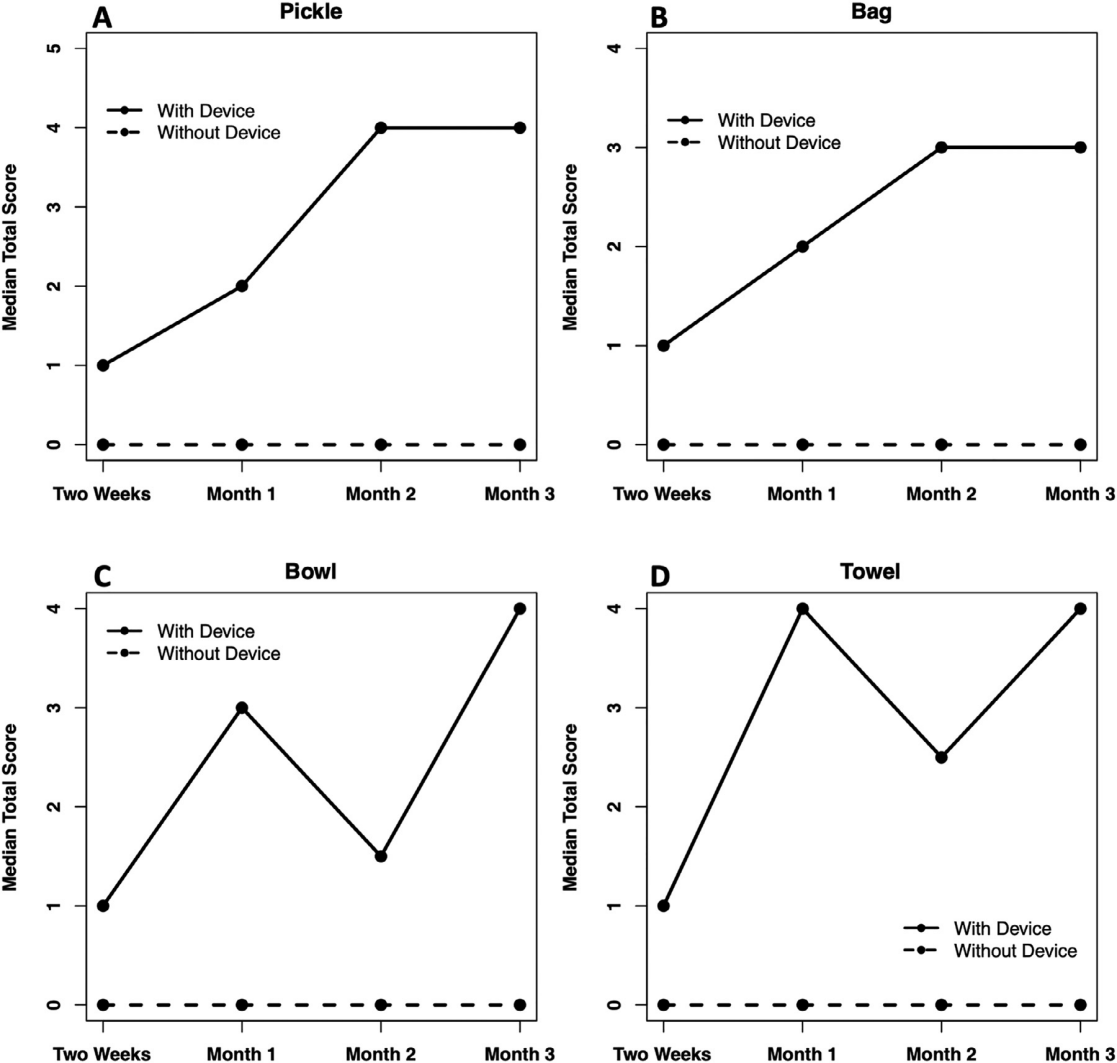
Median total score that participants successfully completed for the tasks over the time points. (A) Pickle: maximum score of 5, (B) Bag: maximum score of 4, (C) Bowl: maximum score of 4, and (D) Towel: maximum score of 4.

When comparing task success over time from 2-Weeks to Month-3, the median total score increased w/MyoPro and did not increase w/o-MyoPro (fig 3, supplemental table S2, available online only at http://www.archives-pmr.org/). In addition, participants had a higher median total completion score w/MyoPro as compared with w/o-MyoPro at each time point.

With the MyoPro, participants had a higher probability of completing the components of the tasks. Table 3 shows that, with exception to Towel Component 1 (adjusted *P*=.18), task completion w/MyoPro was associated with the successes of all task components, compared with w/o-MyoPro (odds ratios ranged from 10.8 to 129,374.1, adjusted *P*’s<.05). We failed to find that the “with MyoPro” effects compared with “without MyoPro” varied across the 3 time points. No significant interaction effects between time point and w/wo MyoPro status were found after multiple testing adjustments (adjusted *P*’s>.05, supplemental table S3, available online only at http://www.archives-pmr.org/).

**Table 3:** Main effect of each task component with the MyoPro

	OR (95% CI)	P*	Adj *P*†
**Pickle P1**	42046.9 (75.5, 23,406,527.7)	.00096	.0048
**Pickle P2**	27.7 (7.4, 103.7)	8.44E-07	<.0001
**Pickle P3**	16.7 (4.9, 57.2)	7.45E-06	<.0001
**Pickle P4**	16.966 (4.273, 67.363)	5.71E-05	.0003
**Pickle P5**	160.1 (5.9, 4333.9)	.0026	.013
**Bag P1**	12211.5 (45.8, 3,256,795.3)	.00096	.0038
**Bag P2**	207.4 (19.4, 2221.6)	1.04E-05	<.0001
**Bag P3**	122.3 (10.4, 1440.6)	.00013	.00053
**Bag P4**	10.8 (2.3, 50.5)	.0025	.0099
**Bowl P1**	129374.1 (25.6, 653,660,663.8)	.0068	.027
**Bowl P2**	93.2 (13.1, 663.4)	5.95E-06	<.0001
**Bowl P3**	22.1 (5.8, 84.9)	6.56E-06	<.0001
**Bowl P4**	15.9 (3.6, 71.1)	.00029	.0012
**Towel P1**	4,980,565.5 (1.5, 16,237,911,476,843.2)	.044	.18
**Towel P2**	214.0 (22.643, 2023.1)	2.84E-06	<.0001
**Towel P3**	21.8 (5.722, 83.3)	6.38E-06	<.0001
**Towel P4**	25.6 (5.8, 113.5)	2.00E-05	<.0001

NOTE. “P#” stands for each component of each task.

Abbreviations: OR, odds ratio

⁎ Results of longitudinal mixed logistic models with predictors of time variable, w/wo MyoPro status, adjusted for the corresponding completion status of the first component in each task with the MyoPro at the first time point (2 weeks after receiving the MyoPro) and other chosen covariates.

† Holm-Bonferroni adjusted *P* values.

## Discussion

For individuals with hemiparesis after stroke, the MyoPro provides the ability to accomplish I/ADLs with the paretic side that they were previously unable to accomplish. Participants completed the tasks quicker and had a higher probability of successful completion w/MyoPro as compared with w/o-MyoPro. Comments of fulfillment were expressed after achieving tasks w/MyoPro compared with comments of frustration when attempting tasks w/o-MyoPro. The significance of the greater success in completing the tasks w/MyoPro than without suggests a clinically meaningful outcome. The inability to accomplish these tasks w/o-MyoPro remained fairly stable with little change within the first 3 months, which clearly illustrates the participants’ continuing inability to accomplish I/ADLs. Thus, use of the MyoPro is likely to increase activity participation and decrease disability, thereby filling a critical need for individuals with chronic motor impairment post-stroke.

Predicting who would be the best candidates for success using the MyoPro, as with any intervention, is very important. In this study, individuals who successfully completed the first component of each task w/MyoPro at 2-Weeks may be more likely to succeed with using the MyoPro for I/ADL completion, meaning that early success w/MyoPro may make it easier to learn further functional improvements (supplemental table S4, available online only at http://www.archives-pmr.org/).

In this study, individuals were learning new coordination patterns, such as volitionally controlling the MyoPro, relaxing co-contractions, and simultaneously activating their Biceps/Triceps muscle while activating forearm Flexors/Extensors during activities. We examined proficiency by using the completion time and scores in each task. It would be anticipated that completion times at successive time points would be faster than the prior time points. However, our data showed that the path to proficiency was not a continuously improving profile of functional ability. Individuals typically begin using their MyoPro with Biceps mode at the Elbow (neuro re-education of Biceps muscle activation/relaxation) and Close mode at the Hand (neuro re-education of forearm flexor muscle activation/relaxation). Once successful in these modes, users progress to MyoPro modes that activate/relax the opposite muscles. Because users are likely balancing speed-accuracy tradeoffs as they learn new coordination patterns w/MyoPro, there can be a non-linear trajectory of improvement in completing the tasks, as seen in the Bowl and Towel tasks. Once different modes are learned and adequate control of agonist-antagonist muscles with minimal co-contraction has been gained, users can achieve an even greater level of MyoPro proficiency. The time needed to gain MyoPro proficiency may be based on the interaction of their unique impairment and the complexity of each tasks’ components.

After 3 months of using the MyoPro, participants reported improved confidence and anecdotally reported completing real-world activities around the home (table 4). These functional gains occurred in individuals with limited motor control over 6 months post-stroke, a population that typically has a poor ability to recover motor skills and often remains significantly impaired for life.^21^, ^22^, ^23^ Increasing individuals’ functional abilities could positively affect their mental health and level of post-stroke depression.^24^ Future research should measure MyoPro users’ daily function (I/ADLs) and engagement in therapy in addition to the simulated battery of tasks.

**Table 4:** Self-reported improvements in arm impairment and functional use

	Examples of Self-reported Changes
**With MyoPro**	Hold and transfer objects (water bottle, put socks away in a drawer, independently carry dishes on tray from dining table to kitchen sink) Fold towels; sweep Carry grocery bag Make muffins (hold bowl with MyoPro side, use MyoPro side to hold spatula while scraping bowl)
**Without MyoPro**	Hand is looser and rests open Carry objects with 2 hands (laundry basket) Open building and car doors Carry bag in affected hand to keep unaffected hand free to hold stair rail Hold bowl while preparing food Use affected hand to stabilize dishes when washing in the kitchen sink

### Study limitations

Although the results demonstrate that participants can be successful in completing I/ADLs with their MyoPro, the sample size was small and more time using the MyoPro may be needed for individuals to be successful with all components of each task. The interaction between the w/wo conditions and time variable did not show significance after adjusting for multiple testing. This may also be due to the small sample size and the short time frame. As an observational study, the research team did not prescribe any particular training, therapy, or specific modes other than what the participants would do on their own. Therefore, we do not know how much or what kind of therapy/training participants received w/MyoPro. It is possible that individuals could show greater gains in ability to complete the functional tasks with targeted training.

In addition, this outcome measure has not been validated and may not accurately represent the participants’ progress in MyoPro proficiency. We chose this battery of tasks as it reflects the gross motor capabilities of the MyoPro and because we were unable to provide hands-on assessment nor have therapist involvement due to this being a nationwide observational study. There is also the possibility for rater variability or bias even though all raters were trained the same. The tasks require the users to remain seated, but there was variability in table and chair heights available in participants’ homes, which may or may not have affected the probability of success. Despite these variabilities, the functional tasks used in this study provide an alternative approach for evaluating user proficiency and capabilities in a way that can be representative of the MyoPro's functionality.

## Conclusions

The MyoPro has the potential to help individuals after stroke be more successful at completing I/ADLs when using a MyoPro. Higher probability of success and reduced time to complete functional tasks were observed w/MyoPro as compared with w/o-MyoPro. The MyoPro's effect should be studied over a longer time frame to determine the optimal training time for functional use, in how people use the MyoPro in daily life and for which tasks it promotes successful completion and which it does not, and with larger samples to help delineate clear variables that predict increase in function w/MyoPro.

## Suppliers

^a^MyoPro; Myomo, Inc, Boston, MA.

^b^SAS software; SAS Institute, Inc, Version 9.4, Cary, NC.

^c^R statistical software version 4.2.1; R Core Team.

## Appendix Supplementary materials

Image, application 1

Image, application 2

## Footnotes

Supplementary material associated with this article can be found, in the online version, at doi:10.1016/j.arrct.2023.100279.
