# Supplementary material for: Myoelectric Arm Orthosis Assists Functional Activities: A 3-Month Home Use Outcome Report
Source: Arch Rehabil Res Clin Transl. 2023 Jul 13;5(3):100279. doi: 10.1016/j.arrct.2023.100279 (PMC10517359; doi:10.1016/j.arrct.2023.100279)
Supplement: Supplementary file 2 [file mmc2.docx]

**Detailed Inclusion/Exclusion Criteria**

Participants were recruited and included if they (a) were 18 years of age or older; (b) had an upper limb impairment caused by any stroke; (c) were medically stable; (d) were in the process of being fit with a MyoPro as a first-time user (d) had at least trace muscle activation in the elbow and wrist flexors and extensors as measured by a 1/5 on a manual muscle test (Medical Research Council Scale for Muscle Strength) at target joints; (e) had the minimum microvolt EMG threshold to operate the MyoPro such that the individual can sustain the EMG signals above the threshold for two full seconds (both at elbow and hand) for a minimum of three times within three minutes; (f) had passive range of motion within 5 degrees of terminal range for finger and elbow flexion and extension; (g) had passive range of motion within 5 degrees of terminal range for wrist flexion and at least neutral for wrist extension; (h) had at least 30 degrees of active range of motion for shoulder flexion or shoulder abduction; (i) were able to support the weight of the MyoPro; (j) were able to tolerate functional tasks for 20 minutes with intermittent rests without excessive fatigue; (k) had intact cognition; (l) were able to clearly and verbally communicate in the English language; (m) had a wireless internet connection to participate in telehealth sessions; and (n) were intending and willing to attend therapy within four weeks of getting fit and trained with a MyoPro.

Individuals were excluded if they (a) had a body weight above 235 lbs; (b) had upper limb measurements that prevented correct fit of the orthosis; (c) had fixed upper limb contractures on the affected side; (d) were unable to have full passive finger opening when the wrist was in neutral; (e) had severe spasticity or tone defined as greater than a 2 at the wrist or fingers, or 3 or higher at the elbow on the Modified Ashworth Spasticity Scale; (f) had severe shoulder subluxation (greater than one finger with significant pain) or shoulder dislocation; (g) had passive shoulder range of motion less than 45 degrees in flexion and abduction; had excessive pain, hypersensitivity, or skin issues in the hand, arm(s), or shoulder(s) that would prevent wearing the MyoPro; (h) had severe cognitive or psychiatric problems that might be contraindicated for training and safe MyoPro use; (i) had bilateral upper limb impairment; was pregnant; and any other conditions (e.g., history of neurological disorder other than CVA) or circumstances that would preclude safe and/or effective participation, including severe sensory deficits, skin conditions, and/or other sequelae that may be contraindicated for myoelectric MyoPro use.

**Detailed Functional Tasks Instructions**

All tasks began with the participant’s arms and hands off the table. The first component of each task is to bring both arms and hands to the table with the hands in an open position. To receive a score of 1 (able to achieve), participants needed to open the fingers adequately such that the individual would not need to force the objects into the hand when attempting to grasp. This may be fingers in full extension or may have the fingers open enough that a soda can could fit in the gap between the fingers and thumb. When placing the hands on the table, participants could position the hands in any position, but the raters needed to be able to observe that the participant was actively opening the hands (rather than using the tabletop to extend the fingers).

Participants were asked to not use their unaffected arm to assist the affected arm in completing the task. For example, when bringing the arm to the table, participants were informed that they should bring the MyoPro side to the table as independently as possible. When grasping an object, participants were directed to avoid shoving the object into their hand but to instead focus on opening the hand prior to grasping the object. When lifting an object off the table, participants were directed to avoid using the non-MyoPro side to assist in raising the MyoPro side off the table.

When completing the task with the MyoPro, participants were allowed to select the MyoPro mode that they preferred to use for the task and could reposition their wrist in the MyoPro as desired. The MyoPro has three modes at the elbow: Biceps, Triceps, and Dual. Similarly, there are three modes at the hand: Close, Open, and Dual. In Biceps mode, the biceps muscle needs to activate above the user-specified threshold to flex the elbow and relax below the threshold to extend the elbow. In Triceps mode, the triceps muscle needs to activate above the user-specified threshold to extend the elbow and relax below the threshold to flex the elbow. In Dual mode at the elbow, the biceps muscle needs to activate above the biceps’ user-specified threshold to flex the elbow and the triceps muscle needs to activate above the triceps’ user-specified threshold to extend the elbow. In Close mode, the forearm flexor muscle needs to activate above the user-specified threshold to flex the fingers and relax below the threshold to extend the fingers. In Open mode, the forearm extensor muscle needs to activate above the user-specified threshold to extend the fingers and relax below the threshold to flex the fingers. In Dual mode at the hand, the forearm flexor muscle needs to activate above the forearm flexor’s user-specified threshold to flex the fingers and the forearm extensor muscle needs to activate above the forearm extensor’s user-specified threshold to extend the fingers.

All tasks were completed in a seated position. Each participant used a table and chair that was available in their own home. The chair may or may not have had armrests, depending on what the participant had available. The height of the table was determined by the table that each participant had available for use.

The battery of tasks was evaluated by one rater at each session. There were three raters who received training on the battery of tasks and conducted the sessions (S.C., N.H., M.G.). Two raters (S.C., M.G.) designed the study and trained together during the development and then all three raters were trained together. Practice sessions were conducted amongst the raters to verify alignment in scoring and timing. A component of training was that the raters trained together until the scores and times aligned.

The following are details on the instructions provided to participants during the tasks.

“Pickle: Move object to mouth” (Figure 1A)

ADL examples: meal preparation, take a bite, take a drink, brush teeth, wipe nose, wash face

Setup: A plastic pickle (35 g, L: 26.5 cm, with circumference of 11.0 cm at midpoint) was placed horizontally in front of the participant on the table. A plastic knife was placed vertically on the non-MyoPro side.

Component 1: The participant started by bringing both arms and hands to the table, with their hands open. The participant was required to bring the MyoPro side to the table independently without assistance from the non-MyoPro side. (start timing with stopwatch when participant initiated movement toward the table; stop timing when the hands were functionally open)

Component 2: With the affected hand, the participant grasped the plastic pickle on the table for two seconds. The participant was allowed to reposition the pickle to allow grasp, which could include moving the pickle closer to the affected hand or lifting the pickle off the table to a height that the MyoPro side can feasibly grasp. (start timing with stopwatch when participant initiated movement toward the pickle; stop timing once the pickle was grasped for the duration of the two second count)

Component 3: While holding the pickle, the participant used the unaffected hand to simulate cutting the pickle with a plastic knife for two seconds. The participant needed to continue holding the pickle for the entire duration of two seconds; if the participant lost the grasp of the pickle, the two second count restarted after the pickle was held again and cutting was simulated. (start timing with stopwatch when participant picked up the knife; stop timing once the two second count while cutting was complete)

Component 4: The participant continued to hold the pickle and brought the pickle “to their mouth” to hold for two seconds. The participant needed to bring their entire arm off the table. If the participant lost the grasp of the pickle before two seconds, the participant needed to regrasp the pickle and bring the pickle to their mouth to restart the two second count. (start timing with stopwatch when participant initiated movement toward the mouth; stop timing after the two second count while holding was complete)

Component 5: The participant then lowered the pickle to the table, released the grasp on the pickle by opening the hand, and ended with both arms and hands in an open position on the table. (start timing with stopwatch when participant initiated movement toward the table; stop timing when the hands were functionally open)

Maximum possible score is 5. Maximum possible time allowed is 225 seconds.

“Bag: Hold object in space” (Figure 1B)

ADL examples: carry shopping bag, carry laundry basket, move item off of lap

Setup: A bean bag weight (109 g) was placed inside of a paper bag that had two handles at the top. (H: 25.5 cm, W: 20.3 cm, D: 12.1 cm, with handles that are 10.5 cm tall) The paper bag was set on the participant’s lap. If unable to keep the bag on their lap in a stable position, the participant was allowed to set the bag aside on the table to start and then return the paper bag to be within reach when attempting Component 2.

Component 1: The participant started by bringing both arms and hands to the table, with their hands open. The participant was required to bring the MyoPro side to the table independently without assistance from the non-MyoPro side. (start timing with stopwatch when participant initiated movement toward the table; stop timing when the hands were functionally open)

Component 2: With the affected hand, the participant grasped the handles of the paper bag for two seconds. The participant was allowed to reposition the paper bag handles, if necessary, and could use the unaffected side to hold the handles stable while the affected hand grabbed the handles. (start timing with stopwatch when participant initiated movement toward the bag handles; stop timing once the bag handles have been held for the two second count)

Component 3: While holding the bag handles with the affected hand, the participant lifted the paper bag off their lap and held the bag “in space” for two seconds. The participant needed to bring their entire arm off the table. At minimum, the participant needed to lift the bag off the lap such that the bag was no longer in contact with the lap and was being fully held by their hand. If the participant did not keep a closed grasp on the bag handles for the two second count, the participant needed to regain a closed grasp and the two second count would restart. (start timing with stopwatch when participant initiated movement off lap; stop timing when the bag has been held in space for the two second count)

Component 4: The participant then lowered the paper bag to the lap, released the grasp on the bag handles by opening the hand, and ended with both arms and hands in an open position on the table. (start timing with stopwatch when participant initiated movement toward the table; stop timing when the hands are functionally open)

Maximum possible score is 4. Maximum possible time allowed is 180 seconds.

“Bowl: Stabilize object” (Figure 1C)

ADL examples: cook, meal preparation, hold a container/bag while filling it, dry dishes, simple housekeeping, basic hobbies

Setup: A plastic mixing bowl that had a side handle (13.0 cm tall, with 20.0 cm diameter and 8.0 cm long handle) was placed in front of the participant on the table, with the handle oriented towards the MyoPro side. A bean bag weight (109 g) was placed inside the bowl, and a plastic mixing spoon (28.0 cm long) was set on the table on the non-MyoPro side.

Component 1: The participant started by bringing both arms and hands to the table, with their hands open. The participant was required to bring the MyoPro side to the table independently without assistance from the non-MyoPro side. (start timing with stopwatch when participant initiated movement toward the table; stop timing when the hands were functionally open)

Component 2: With their affected hand, the participant grasped the handle of the plastic mixing bowl for two seconds. If the bowl was sliding around on the table, the participant was allowed to use the non-MyoPro side to keep the bowl from moving. (start timing with stopwatch when participant initiated movement toward the bowl handle; stop timing once the bowl handle has been held for the two second count)

Component 3: While “stabilizing” the mixing bowl with the affected hand, the participant used the mixing spoon held in the unaffected hand to stir the bean bag inside the bowl for five seconds. If the grasp of the bowl handle was lost before five seconds, the participant was required to regrasp the bowl handle and the five second count restarted. (started timing with stopwatch when participant picked up the spoon; stopped timing once the five second count was complete)

Component 4: The participant then placed the mixing spoon on the table, released the grasp on the mixing bowl handle by opening the hand, and ended with both arms and hands in an open position on the table. (started timing with stopwatch when rater cued participant to release the bowl; stopped timing when the hands were functionally open)

Maximum possible score is 4. Maximum possible time allowed is 180 seconds.

“Towel: Move object to a new location” (Figure 1D)

ADL examples: simple clean up, pick up laundry/drop into laundry basket, pick up personal care/grooming items

Setup: A light cloth towel (24 g, H: 35 cm, W: 30.0 cm, 0.3 cm thick) was placed in front of the participant on the table. The participant could choose to have the towel bunched up or flat.

Component 1: The participant started by bringing both arms and hands to the table, with their hands open. The participant is required to bring the MyoPro side to the table independently without assistance from the non-MyoPro side. (started timing with stopwatch when participant initiated movement toward the table; stopped timing when the hands were functionally open)

Component 2: With their affected hand, the participant grasped the towel for two seconds. If needed, the participant was allowed to use the non-MyoPro side to reposition the towel in order for their MyoPro side to adequately grasp the towel, such as moving the towel closer to the MyoPro side or lifting the towel slightly off the table to be within reach of the thumb and index/middle fingers (e.g., three jaw chuck grasp with the MyoPro). (started timing with stopwatch when participant initiated movement toward the towel; stopped timing once the towel was held for the two second count)

Component 3: While continuing to hold the towel with the affected hand, the participant was asked to lift the towel off the table towards the affected side by either turning their body or externally rotating their shoulder and to hold the towel in this “new location” for two seconds. The participant needed to remove their entire arm and the towel off the table. If the grasp of the towel was lost before two seconds, the participant was required to regrasp the towel to hold in this “new location” and the two second count restarted. (started timing with stopwatch when participant initiated movement off the table; stopped timing after the towel was held in space for the two second count)

Component 4: The participant then released the towel by opening the hand to let the towel drop to the ground, returned facing forward if the body was turned, and ended with both arms and hands in an open position on the table. (started timing with stopwatch when participant initiated movement to the table; stopped timing when the hands were functionally open)

Maximum possible score is 4. Maximum possible time allowed is 180 seconds.

*Supplemental Table 1: Interaction effect between the time for each component of each task and when completed with the MyoPro*

*“P#” stands for each component of each task.*

|  | **OR(95%CI)** | **p*** | **adj.p**^†^ |
| --- | --- | --- | --- |
| **Pickle P1** |  |  |  |
| Month 1:WWO | 38.6 (0.1,11757.1) | 0.21 | 1.00 |
| Month 2:WWO | 113.3 (0.3,40687.5) | 0.12 | 0.58 |
| Month 3:WWO | 6326130019.7 (0,Inf) | 0.99 | 1.00 |
| **Pickle P2** |  |  |  |
| Month 1:WWO | 3.9 (0.2,65.5) | 0.34 | 1.00 |
| Month 2:WWO | 12.1 (0.5,304.2) | 0.13 | 0.64 |
| Month 3:WWO | 15.4 (0.7,319.8) | 0.08 | 0.39 |
| **Pickle P3** |  |  |  |
| Month 1:WWO | 3 (0.2,49.8) | 0.45 | 1.00 |
| Month 2:WWO | 11 (0.5,268.3) | 0.14 | 0.71 |
| Month 3:WWO | 9 (0.5,167.4) | 0.14 | 0.70 |
| **Pickle P4** |  |  |  |
| Month 1:WWO | 4.7 (0.2,119) | 0.35 | 1.00 |
| Month 2:WWO | 28.1 (0.8,1032.7) | 0.07 | 0.35 |
| Month 3:WWO | 45.1 (1.4,1446.9) | 0.03 | 0.16 |
| **Pickle P5** |  |  | 0.00 |
| Month 1:WWO | 6.2 (0,Inf) | 1.00 | 1.00 |
| Month 2:WWO | 8.6 (0,Inf) | 1.00 | 1.00 |
| Month 3:WWO | 0 (0,Inf) | 1.00 | 1.00 |
| **Bag P1** |  |  |  |
| Month 1:WWO | 6.1 (0.1,655.8) | 0.45 | 1.00 |
| Month 2:WWO | 12.5 (0.1,2465) | 0.35 | 1.00 |
| Month 3:WWO | 6.6 (0.1,716.4) | 0.43 | 1.00 |
| **Bag P2** |  |  |  |
| Month 1:WWO | 7.9 (0.2,348.7) | 0.29 | 1.00 |
| Month 2:WWO | 175.2 (1.3,24143.6) | 0.04 | 0.16 |
| Month 3:WWO | 15.8 (0.3,779.5) | 0.16 | 0.66 |
| **Bag P3** |  |  |  |
| Month 1:WWO | 6 (0.1,394.9) | 0.40 | 1.00 |
| Month 2:WWO | 80.2 (0.6,11680.5) | 0.08 | 0.34 |
| Month 3:WWO | 7.8 (0.1,447.6) | 0.32 | 1.00 |
| **Bag P4** |  |  |  |
| Month 1:WWO | 154.1 (1.2,19250.6) | 0.04 | 0.16 |
| Month 2:WWO | 6874047799.1 (0,Inf) | 0.99 | 1.00 |
| Month 3:WWO | 74.5 (0.8,7084) | 0.06 | 0.25 |
| **Bowl P1** |  |  |  |
| Month 1:WWO | 374268158493.9 (0,Inf) | 1.00 | 1.00 |
| Month 2:WWO | 1779.1 (2.6,1232891) | 0.02 | 0.10 |
| Month 3:WWO | 235631212476.4 (0,Inf) | 1.00 | 1.00 |
| **Bowl P2** |  |  |  |
| Month 1:WWO | 5.7 (0.2,203.3) | 0.34 | 1.00 |
| Month 2:WWO | 6.4 (0.1,303.6) | 0.35 | 1.00 |
| Month 3:WWO | 95.2 (1.5,6100.3) | 0.03 | 0.13 |
| **Bowl P3** |  |  |  |
| Month 1:WWO | 20.2 (0.9,462.6) | 0.06 | 0.24 |
| Month 2:WWO | 18.4 (0.6,575.9) | 0.10 | 1.00 |
| Month 3:WWO | 57.6 (2,1629.1) | 0.02 | 0.07 |
| **Bowl P4** |  |  |  |
| Month 1:WWO | 26.3 (0.7,1058.9) | 0.08 | 0.33 |
| Month 2:WWO | 174591649.5 (0,Inf) | 1.00 | 1.00 |
| Month 3:WWO | 61.7 (1.9,1988.7) | 0.02 | 0.08 |
| **Towel P1** |  |  |  |
| Month 1:WWO | 71579.5 (0.2,25072246687.3) | 0.09 | 0.34 |
| Month 2:WWO | 51.5 (0,246313.6) | 0.36 | 1.00 |
| Month 3:WWO | 62.8 (0,140232.9) | 0.29 | 11.00 |
| **Towel P2** |  |  |  |
| Month 1:WWO | 20.1 (0.5,884.4) | 0.12 | 0.48 |
| Month 2:WWO | 8.3 (0.2,430.8) | 0.29 | 1.00 |
| Month 3:WWO | 29.5 (0.6,1352) | 0.08 | 0.33 |
| **Towel P3** |  |  |  |
| Month 1:WWO | 8.2 (0.4,162.4) | 0.17 | 0.67 |
| Month 2:WWO | 6.6 (0.3,170.1) | 0.26 | 1.00 |
| Month 3:WWO | 18.8 (0.8,441.9) | 0.07 | 0.27 |
| **Towel P4** |  |  |  |
| Month 1:WWO | 22.6 (0.8,658) | 0.07 | 0.28 |
| Month 2:WWO | 209258463.1 (0,Inf) | 0.97 | 1.00 |
| Month 3:WWO | 18.4 (0.8,432) | 0.07 | 0.28 |

* Results of longitudinal mixed logistic models with predictors of time variable, w/wo MyoPro status, interaction term between time and w/wo MyoPro status adjusted for the corresponding completion status of the first component in each task with the MyoPro at the first time point (2-Weeks after receiving the MyoPro) and other chosen covariates.

^†^ Holm-Bonferroni adjusted p-values

*Supplemental Table 2: Mean (SD) time [seconds] to complete each component of the tasks*

*“P#” stands for each component of each task.*

|  |  | **2-Weeks** | | **Month 1** | | **Month 2** | | **Month 3** | |
| --- | --- | --- | --- | --- | --- | --- | --- | --- | --- |
|  |  | **w** | **wo** | **w** | **wo** | **w** | **wo** | **w** | **wo** |
| **Pickle** | **P1** | 22.5 (19.6) | 38.3 (15.5) | 13.8 (15.7) | 40.4 (13.4) | 15.6 (15.9) | 42.5 (10.0) | 11.1 (8.5) | 40.1 (13.9) |
|  | **P2** | 33.6 (16.3) | 41.0 (11.7) | 28.5 (16.9) | 41.1 (11.3) | 22.5 (17.8) | 42.6 (9.8) | 22.3 (15.8) | 40.4 (13.0) |
|  | **P3** | 34.8 (16.8) | 40.8 (12.4) | 29.5 (18.3) | 40.6 (12.9) | 23.2 (18.9) | 43.7 (5.2) | 19.1 (18.4) | 40.2 (13.6) |
|  | **P4** | 39.0 (13.9) | 40.8 (12.3) | 31.9 (18.3) | 40.7 (12.6) | 27.7 (18.2) | 42.8 (8.8) | 18.9 (18.3) | 40.6 (12.3) |
|  | **P5** | 41.5 (10.1) | 45.0 (0.0) | 38.8 (12.2) | 45.0 (0.0) | 36.1 (15.9) | 45.0 (0.0) | 32.6 (15.5) | 40.7 (12.1) |
|  | **Total** | 171.4 (64.4) | 205.8 (49.3) | 142.5 (65.1) | 207.7 (50.3) | 125.1 (62.8) | 216.6 (33.8) | 103.9 (58.1) | 202.0 (64.9) |
|  |  | | | | | | | | |
| **Bag** | **P1** | 22.1 (18.4) | 40.6 (12.8) | 16.5 (16.0) | 40.6 (12.9) | 14.8 (15.7) | 42.7 (9.2) | 13.9 (15.5) | 40.0 (14.1) |
|  | **P2** | 34.2 (15.8) | 41.1 (11.6) | 28.1 (16.8) | 41.2 (11.2) | 25.8 (15.5) | 42.9 (8.2) | 26.9 (16.4) | 40.3 (13.3) |
|  | **P3** | 33.8 (18.0) | 40.6 (13.0) | 27.5 (19.8) | 40.7 (12.6) | 22.9 (20.1) | 42.7 (9.2) | 22.4 (20.6) | 40.2 (13.5) |
|  | **P4** | 43.4 (6.8) | 40.8 (12.3) | 34.3 (15.1) | 40.8 (12.2) | 34.6 (16.1) | 45.0 (0.0) | 34.1 (15.6) | 40.6 (12.5) |
|  | **Total** | 133.5 (47.0) | 163.0 (49.7) | 106.3 (52.5) | 163.2 (48.9) | 98.1 (53.3) | 173.3 (26.8) | 97.3 (52.9) | 161.1 (53.3) |
|  |  | | | | | | | | |
| **Bowl** | **P1** | 22.8 (19.3) | 40.5 (13.1) | 8.4 (10.2) | 40.7 (12.5) | 13.5 (15.9) | 42.6 (9.8) | 8.2 (6.1) | 38.1 (15.4) |
|  | **P2** | 36.9 (13.1) | 40.8 (12.3) | 23.9 (17.8) | 40.7 (12.6) | 29.6 (17.0) | 42.7 (9.2) | 18.9 (16.3) | 40.5 (12.6) |
|  | **P3** | 41.1 (9.3) | 41.2 (11.0) | 22.8 (17.8) | 41.2 (11.2) | 29.1 (18.6) | 43.6 (5.5) | 21.2 (16.9) | 40.6 (12.3) |
|  | **P4** | 41.3 (10.5) | 40.9 (11.8) | 34.1 (15.9) | 43.2 (7.5) | 36.4 (15.6) | 45.0 (0.0) | 28.4 (19.1) | 40.2 (13.5) |
|  | **Total** | 142.1 (41.9) | 163.4 (48.3) | 89.2 (47.8) | 165.8 (41.8) | 108.6 (54.4) | 173.9 (24.5) | 76.7 (45.9) | 159.5 (52.2) |
|  |  | | | | | | | | |
| **Towel** | **P1** | 23.5 (19.2) | 40.6 (13.0) | 11.0 (13.6) | 40.6 (12.9) | 14.6 (15.8) | 42.5 (10.0) | 10.9 (13.5) | 40.0 (14.1) |
|  | **P2** | 32.5 (16.2) | 40.8 (12.3) | 27.8 (13.9) | 40.8 (12.3) | 26.0 (17.7) | 43.4 (6.5) | 18.9 (14.8) | 40.2 (13.5) |
|  | **P3** | 35.8 (17.1) | 40.7 (12.5) | 22.6 (18.8) | 40.7 (12.5) | 27.5 (18.5) | 42.7 (9.2) | 17.4 (16.8) | 40.5 (12.8) |
|  | **P4** | 37.8 (13.6) | 40.8 (12.3) | 26.0 (17.7) | 42.8 (9.4) | 34.2 (16.7) | 45.0 (0.0) | 21.6 (16.8) | 40.5 (12.8) |
|  | **Total** | 129.6 (55.7) | 162.8 (50.0) | 87.3 (53.0) | 164.8 (44.8) | 102.3 (57.9) | 173.6 (25.8) | 68.8 (51.7) | 161.2 (53.1) |

*Supplemental Table 3: Number (%) of participants that succeeded in each component of the tasks*

*“P#” stands for each component of each task.*

|  | **2-Weeks** | | **Month 1** | | **Month 2** | | **Month 3** | |
| --- | --- | --- | --- | --- | --- | --- | --- | --- |
|  | **w** | **wo** | **w** | **wo** | **w** | **wo** | **w** | **wo** |
| **Pickle P1** | 11 ( 64.7 ) | 3 ( 16.7 ) | 14 ( 82.4 ) | 2 ( 11.1 ) | 14 ( 87.5 ) | 1 ( 6.2 ) | 16(100) | 2 ( 11.8 ) |
| **Pickle P2** | 6 ( 35.3 ) | 2 ( 11.1 ) | 10 ( 58.8 ) | 2 ( 11.1 ) | 11 ( 68.8 ) | 1 ( 6.2 ) | 13 ( 81.2 ) | 2 ( 11.8 ) |
| **Pickle P3** | 5 ( 29.4 ) | 2 ( 11.1 ) | 8 ( 47.1 ) | 2 ( 11.1 ) | 10 ( 62.5 ) | 1 ( 6.2 ) | 11 ( 68.8 ) | 2 ( 11.8 ) |
| **Pickle P4** | 3 ( 17.6 ) | 2 ( 11.1 ) | 6 ( 35.3 ) | 2 ( 11.1 ) | 9 ( 56.2 ) | 1 ( 6.2 ) | 11 ( 68.8 ) | 2 ( 11.8 ) |
| **Pickle P5** | 2 ( 11.8 ) | 0 ( 0.0 ) | 4 ( 23.5 ) | 0 ( 0.0 ) | 4 ( 25.0 ) | 0 ( 0.0 ) | 7 ( 43.8 ) | 2 ( 11.8 ) |
|  | | | | | | | | |
| **Bag P1** | 12 ( 70.6 ) | 2 ( 11.1 ) | 14 ( 82.4 ) | 2 ( 11.1 ) | 13 ( 81.2 ) | 1 ( 6.2 ) | 14 ( 87.5 ) | 2 ( 11.8 ) |
| **Bag P2** | 6 ( 35.3 ) | 2 ( 11.1 ) | 10 ( 58.8 ) | 2 ( 11.1 ) | 12 ( 75.0 ) | 1 ( 6.2 ) | 11 ( 68.8 ) | 2 ( 11.8 ) |
| **Bag P3** | 5 ( 29.4 ) | 2 ( 11.1 ) | 8 ( 47.1 ) | 2 ( 11.1 ) | 9 ( 56.2 ) | 1 ( 6.2 ) | 9 ( 56.2 ) | 2 ( 11.8 ) |
| **Bag P4** | 1 ( 5.9 ) | 2 ( 11.1 ) | 6 ( 35.3 ) | 2 ( 11.1 ) | 6 ( 37.5 ) | 0 ( 0.0 ) | 6 ( 37.5 ) | 2 ( 11.8 ) |
|  | | | | | | | | |
| **Bowl P1** | 10 ( 58.8 ) | 2 ( 11.1 ) | 15 ( 93.8 ) | 2 ( 11.1 ) | 13 ( 81.2 ) | 1 ( 6.2 ) | 16 (100) | 3 ( 17.6 ) |
| **Bowl P2** | 6 ( 35.3 ) | 2 ( 11.1 ) | 11 ( 68.8 ) | 2 ( 11.1 ) | 8 ( 50.0 ) | 1 ( 6.2 ) | 12 ( 75.0 ) | 2 ( 11.8 ) |
| **Bowl P3** | 3 ( 17.6 ) | 2 ( 11.1 ) | 10 ( 62.5 ) | 2 ( 11.1 ) | 7 ( 43.8 ) | 1 ( 6.2 ) | 11 ( 68.8 ) | 2 ( 11.8 ) |
| **Bowl P4** | 2 ( 11.8 ) | 2 ( 11.1 ) | 6 ( 37.5 ) | 1 ( 5.6 ) | 4 ( 25.0 ) | 0 ( 0.0 ) | 9 ( 56.2 ) | 2 ( 11.8 ) |
|  | | | | | | | | |
| **Towel P1** | 10 ( 58.8 ) | 2 ( 11.1 ) | 14 ( 87.5 ) | 2 ( 11.1 ) | 13 ( 81.2 ) | 1 ( 6.2 ) | 14 ( 87.5 ) | 2 ( 11.8 ) |
| **Towel P2** | 8 ( 47.1 ) | 2 ( 11.1 ) | 12 ( 75.0 ) | 2 ( 11.1 ) | 10 ( 62.5 ) | 1 ( 6.2 ) | 14 ( 87.5 ) | 2 ( 11.8 ) |
| **Towel P3** | 5 ( 29.4 ) | 2 ( 11.1 ) | 10 ( 62.5 ) | 2 ( 11.1 ) | 8 ( 50.0 ) | 1 ( 6.2 ) | 12 ( 75.0 ) | 2 ( 11.8 ) |
| **Towel P4** | 4 ( 23.5 ) | 2 ( 11.1 ) | 9 ( 56.2 ) | 1 ( 5.6 ) | 5 ( 31.2 ) | 0 ( 0.0 ) | 11 ( 68.8 ) | 2 ( 11.8 ) |

*Supplemental Table 4: Effect of completion status of the first component in each task with the MyoPro at the first time point (2-Weeks after receiving the MyoPro).*

*“P#” stands for each component of each task.*

|  | **OR (95%CI)** | **p*** | **adj.p^†^** |
| --- | --- | --- | --- |
| **Pickle P1** | 633.9 (3.5, 115433.6) | 0.015 | 0.26 |
| **Pickle P2** | 6.7 (1.7, 27.1) | 0.0072 | 0.12 |
| **Pickle P3** | 4.8 (1.2, 19.1) | 0.025 | 0.43 |
| **Pickle P4** | 3.8 (0.6, 23.3) | 0.15 | 1.00 |
| **Pickle P5** | 93 (0.6, 13868.6) | 0.076 | 1.00 |
| **Bag P1** | 139.6 (1.3, 14712.6) | 0.038 | 0.64 |
| **Bag P2** | 24.7 (1.2, 525.1) | 0.040 | 0.67 |
| **Bag P3** | 29 (1.4, 589.5) | 0.028 | 0.48 |
| **Bag P4** | 6.8 (1, 48.5) | 0.054 | 0.92 |
| **Bowl P1** | 1362.8 (3.5, 525487.5) | 0.018 | 0.30 |
| **Bowl P2** | 33.5 (5.1, 220.7) | 0.00026 | 0.0044 |
| **Bowl P3** | 9.2 (2.5, 34.5) | 0.00099 | 0.02 |
| **Bowl P4** | 6.6 (1, 41.9) | 0.044 | 0.75 |
| **Towel P1** | 3954.1 (0.7, 21168108.2) | 0.059 | 1.00 |
| **Towel P2** | 21.8 (3.1, 155.3) | 0.0021 | 0.04 |
| **Towel P3** | 3.7 (1, 13.2) | 0.048 | 0.81 |
| **Towel P4** | 2.9 (0.8, 9.8) | 0.094 | 1.00 |

*same model as in Table 3

^†^ Holm-Bonferroni adjusted p-values
